# Prebiotics counteract the morphological and functional changes secondary to chronic cisplatin exposition in the proximal colon of mice

**DOI:** 10.1111/jcmm.18161

**Published:** 2024-03-06

**Authors:** Cristina Biagioni, Chiara Traini, Maria Simonetta Faussone‐Pellegrini, Eglantina Idrizaj, Maria Caterina Baccari, Maria Giuliana Vannucchi

**Affiliations:** ^1^ Research Unit of Histology and Embryology, Department of Experimental and Clinical Medicine University of Florence Florence Italy; ^2^ Section of Physiological Sciences, Department of Experimental and Clinical Medicine University of Florence Florence Italy

**Keywords:** choline acetyl‐transferase (ChAT), Connexin 43, interstitial cells of Cajal, methacholine, mucus secretion, PGP9.5, spontaneous contractile activity

## Abstract

Cisplatin is an antimitotic drug able to cause acute and chronic gastrointestinal side effects. Acute side effects are attributable to mucositis while chronic ones are due to neuropathy. Cisplatin has also antibiotic properties inducing dysbiosis which enhances the inflammatory response, worsening local damage. Thus, a treatment aimed at protecting the microbiota could prevent or reduce the toxicity of chemotherapy. Furthermore, since a healthy microbiota enhances the effects of some chemotherapeutic drugs, prebiotics could also improve this drug effectiveness. We investigated whether chronic cisplatin administration determined morphological and functional alterations in mouse proximal colon and whether a diet enriched in prebiotics had protective effects. The results showed that cisplatin caused lack of weight gain, increase in kaolin intake, decrease in stool production and mucus secretion. Prebiotics prevented increases in kaolin intake, changes in stool production and mucus secretion, but had no effect on the lack of weight gain. Moreover, cisplatin determined a reduction in amplitude of spontaneous muscular contractions and of Connexin (Cx)43 expression in the interstitial cells of Cajal, changes that were partially prevented by prebiotics. In conclusion, the present study shows that daily administration of prebiotics, likely protecting the microbiota, prevents most of the colonic cisplatin‐induced alterations.

## 1 INTRODUCTION

Cisplatin is one of the most widely used platinum derivatives for the treatment of many types of cancer.^1^ Gastrointestinal (GI) side effects (nausea, vomiting, diarrhoea and constipation) are present in up to 100% of the patients treated with this drug; these symptoms appear hours or days after the beginning of the treatment and significantly prejudice the patient's quality of life up to the possibility to preclude therapeutic continuation. GI symptomatology has been attributed to mucosal inflammation. Furthermore, it has been reported that chemotherapy with cisplatin causes chronic neurotoxic damage whose symptoms may persist up to 10 years after discontinuation of the drug.^2^, ^3^ The mechanisms of cisplatin toxicity are multiple and partially known.^3^, ^4^, ^5^ The so‐called *platination* of DNA^6^, ^7^ could explain both the acute damage resulting from a proliferation block in mitotic (i.e. epithelial) cells, and long‐lasting damage in postmitotic cells with high transcriptional activity such as the neurons.^2^, ^3^ Finally, cisplatin acts as an antibiotics on the microbiota^8^ causing dysbiosis^9^ which, added to the damage of the epithelium, enhances the local inflammatory response in a vicious circle that often extends to the whole organism.^10^

It should be noted that the integrity of the microbiota has proved to be essential to enhance the effects of some chemotherapy drugs, cisplatin included.^9^, ^10^, ^11^ Thus, protecting the microbiota could both prevent or reduce chemotherapy toxicity and improve its efficacy. Notably, a layer of mucus secreted by the glandular cells is located between the epithelium and the flora that protects the GI wall from pathogens and toxic luminal molecules and guarantees nutrients to the microbiota^12^ which, in turn, regulates and controls the mucus production/quality.^10^ Experimental investigations in animals have confirmed that cisplatin impairs GI wall integrity.^10^, ^13^, ^14^, ^15^, ^16^ In particular, it causes epithelial damage and dysbiosis followed by a breakdown of the intestinal barrier, activation of the local immune response^2^, ^3^ and mucositis. Until now, however, few information is available on the effect of cisplatin on colonic mucus secretion; recently, we reported that it was significantly decreased in the mouse distal colon.^16^

As expected, cisplatin determines neurotoxicity also in the experimental animals and the severity of the damage was found region‐ and dose‐dependent.^2^, ^4^, ^14^, ^15^, ^16^, ^17^, ^18^ In the mouse and rat stomach and distal colon, a significant decrease in the number of myenteric neurons, an impairment of inhibitory and excitatory neurotransmission^14^, ^15^, ^16^ and loss of glial cells^16^, ^19^, ^20^ were reported in association with reduced GI motility.^14^, ^19^, ^20^

The enteric nervous system regulates the GI function in association and continuity with the interstitial cells of Cajal (ICC).^21^ Three subpopulations of ICC are present in the colon: one at the myenteric plexus, surrounding ganglia and nerve strands (ICC‐MP); a second one in the thickness of the muscle layers (ICC‐IM), oriented parallel to the muscle cells; a third one along the submucosal border of the circular muscle layer (ICC‐SM).^21^, ^22^, ^23^ ICC are able to (i) generate the slow waves (pacemaker activity) responsible for the peristalsis; (ii) integrate the excitatory and inhibitory neurotransmission with the pacemaker activity in order to control peristalsis and (iii) act as stretch receptors recording and responding to local, circumscribed stimuli.^21^, ^24^, ^25^ Consequently, these cells may also be a target of cisplatin toxicity; however, data available in the literature are very few and limited to the stomach and ileum.^19^, ^26^

All of the above information underscores the need to find treatments able to mitigate cisplatin toxicity. Attempts have been made to overcome this problem; unfortunately, while some of the attempted treatments have resulted in reduced efficacy of chemotherapy, others have been followed by otherwise disabling side effects.^3^ Therefore, the ability to control drug toxicity while maintaining antitumor efficacy remains a challenge for researchers and clinicians.

Presently, we studied the effects of chronic cisplatin treatment in the mouse proximal colon, as this is the least studied GI region, evaluating both morphological (i.e. mucosal integrity, mucus secretion, presence of inflammatory cells, myenteric neurons and ICC) and functional (spontaneous muscle mechanical activity) parameters. Since protecting the microbiota could both prevent or reduce chemotherapy toxicity and improve its efficacy, in parallel, we assessed whether the addition of a prebiotic mixture to the diet protected the colon wall from cisplatin‐induced alterations.

## 2 MATERIALS AND METHODS

### 2.1 Animals and treatments

The present research was designed in compliance with the guidelines of the European Communities Council Directive 2010/63/UE and was authorized by the Italian Minister of Health (code: 217/2020). C57BL/6JolaHsd male mice (18–22 g; *n* = 21) were purchased from ENVIGO (S.Pietro al Natisone—UD, Italy). The animals were housed on a 12‐h light/dark cycle under standardized conditions of temperature and humidity with free access to food and water. They were allowed to acclimatize to the animal facility for 1 week. Then they were randomly divided as follows:

*Control group*: untreated mice, *n* = 6, Ctrl;

*Cisplatin group*: mice treated with cisplatin (details below), *n* = 6, Cspl;

*Prebiotics group*: mice fed with an enriched diet with prebiotics (details below), *n* = 4, Prebio;

*Prebiotics‐cisplatin group*: mice fed with enriched diet with prebiotics and treated with cisplatin, *n* = 5, Cspl‐Prebio.

Each group was housed in a separate cage. Mouse's general condition was assessed daily. Specifically, the body weight was measured at the animal's arrival and during the treatment, every 2–4 days. Cisplatin injectable solution (kindly provided by Dr. M. Cecchi, AOU Careggi, Florence, Italy) was intraperitoneally (i.p.) administered twice a week (on Tuesday and Friday) at a dosage of 3 mg/kg. To prevent cisplatin‐induced nephrotoxicity, 0.5 mL of saline solution was injected subcutaneously just before each cisplatin injection,^27^ and the mice, apart from the controls, received the same number of injections. The treatments last 4 weeks. Three days after the last cisplatin injection, the animals were sacrificed.

The prebiotics are a multi‐extract of fibres and plant complexes, mainly composed of inulin/FOS (fructo‐oligosaccharides) that were kindly provided by the Aboca Company [Plantaflor®, Aboca Sansepolcro (AR), Italy]. The dose of prebiotics was 50 mg inulin/FOS/g diet, corresponding to a 5% increase in inulin/FOS in the standard diet; this choice aligns with the literature.^28^, ^29^ The mix of prebiotics will be directly added to the daily diet and maintained for the 4 weeks of treatment. The weight of ingested food was checked daily. The antiemetic kaolin was added to all cages.^30^

### 2.2 Tissue sampling

Full‐thickness samples of proximal (starting 0.5 cm far from the ileo‐caecal junction) colon were taken from each animal, fixed in 4% paraformaldehyde in 0.1 M phosphate‐buffered saline (PBS, pH 7.4) overnight (ON) at 4°C, dehydrated in graded ethanol series, cleared in xylene and embedded in paraffin. Histological transverse (full‐thickness) sections 5 μm thick were cut using a rotary microtome (HistoCore MULTICUT, Leica, Buccinasco, MI, Italy) and collected on normal or positively charged slides, as appropriate.

### 2.3 Histology and histochemistry

The full‐thickness sections were deparaffinized in xylene and re‐hydrated in descending ethanol series up to distilled water. The sections were stained with haematoxylin–eosin to evaluate the tissue architecture and cell infiltrate or with toluidine blue (TB) to evaluate the mucous secretion. For TB staining, the sections were soaked for 10 min in pre‐filtered 0.1% TB in 30% ethanol; washed in distilled water, dehydrated in ascending ethanol and mounted in synthetic resin. All the sections were stained in a single session to minimize artefactual staining differences. Digital images were acquired with a video camera‐equipped microscope (Eclipse 200; Nikon Instruments, Tokyo, Japan) with ×20 objective.

### 2.4 Immunohistochemistry

The paraffin‐embedded sections, once deparaffinized and rehydrated as usual, were treated for antigen retrieval for 20 min at 90–92°C in Tris buffer (10 mM) with EDTA (1 mM, pH 9.0), followed by cooling to room temperature (RT). The sections were then washed in PBS and blocked for 20 min with 5% normal donkey serum (NDS, Jackson Laboratories, Inc.) in PBS. The primary antibodies (Table S1) were diluted in 5% NDS and incubated ON at 4°C. The next day, the sections were incubated for 2 h at RT in the dark with appropriate fluorochrome‐conjugated secondary antibodies diluted 1:333 in 5% NDS. Tissue sections were thoroughly washed in 0.1 M PBS and mounted in an aqueous medium (FluoreGuard Mounting Medium, ScyTek Laboratories Inc., Utah, USA). Double labelling experiments were done with Choline acetyl‐transferase (ChAT) and neuronal nuclei (NeuN) antibodies. After the first incubation as described above, the sections were re‐incubated with the second primary antibody and with the appropriate secondary antibody, following the same procedures. To identify the potential localization of Connexin (Cx) 43 on ICC, sequential sections, 4 μm thick, were collected on slides (4 sections/slide, 2 slides/specimen) in two separate areas, one area containing the first and the third section, the other area containing the second and the fourth section. Each area was bordered with a pap pen, and the two sections of one area were incubated with Cx43 antibody as described above the two sections of the neighbour area were incubated with c‐Kit antibody ON at 4°C. The two immunoreactions were revealed by using the appropriate secondary antibodies diluted 1:333 in 5% NDS, incubated for 2 h, at RT. To exclude the presence of non‐specific immunofluorescence labelling, negative controls were performed by omitting the primary antibody (data not shown). Table S1 summarizes information on primary and secondary antibody sources and used concentrations. Immune reaction was observed and the image of the entire transverse section was acquired with 20× or 60× objectives by an Olympus BX63 fluorescence microscope (Olympus, Italy) or by a Leica Stellaris 5 confocal microscope (Leica Microsystems, Mannheim, Germany) equipped with lasers that emit at 488 or 594 wavelengths to excite green and red fluorescent labels, respectively.

### 2.5 Functional studies: spontaneous mechanical activity

As previously,^31^ full‐thickness circular muscle strips were dissected from colon segments, mounted in double‐jacketed 5 mL organ baths containing Krebs Henseleit solution of the following composition (in mmol/L): 118NaCl, 4.7KCl, 1.2MgSO_4_, 1.2KH_2_PO_4_, 25NaHCO_3_, 2.5CaCl_2_ and 10 glucose, pH 7.4 and bubbled with 95%O_2_–5%CO_2_. Temperature was maintained within a range of 37 ± 0.5°C. One end of each strip was tied to a platinum rod, whereas the other was connected to a force–displacement transducer (FT03; Grass Instrument, Quincy, MA) by a silk thread for continuous recording of isometric tension. The transducer was coupled to a polygraph (7K; Grass Instrument). Strips equilibrated for 1 h under an initial load of 0.2 g. During this period, the preparations underwent repeated and prolonged washes with Krebs–Henseleit solution to prevent the accumulation of metabolites in the organ baths. Smooth muscle contraction was obtained by the addition of methacholine to the bath medium. The interval between two subsequent applications of methacholine was not less than 30 min, during which repeated and prolonged washes of the preparations with Krebs–Henseleit solution were performed.

*Drugs*: the nerve blocker tetrodotoxin (TTX, 1 × 10^−6^ M) and the muscarinic receptors agonist methacholine (2 × 10^−6^ M) were used. The concentrations were those previously used in murine gastrointestinal preparations and proved to be effective.^32^ All drugs were obtained from Sigma (St. Louis, MO, USA). Methacholine solution was freshly prepared while a TTX stock solution was kept stored at −20°C.

### 2.6 Quantitation and statistical analysis

The quantitation of both TB staining and c‐Kit and Cx43 labelling was done by two observers (CB, CT), blind to each other, in the entire transverse section (2 sections/animal; 4 animals/group) of proximal colon. The blind procedure consisted of randomly assigning the specimens a progressive number, from 1 to 16 (4 animals/4 groups), so that the researcher did not know which group each specimen belonged to. At the end of the quantitation, the values obtained from two sections/animals were attributed to the corresponding specimens.

The images were acquired using 20× objective and the entire section was reconstructed to correctly overlapping the shared tracts with appropriate software. Data quantitation was done using ImageJ software (NIH, Bethesda, ML, USA) and the results were expressed as pixel^2^/section. Specifically, for the mucosal area and for the TB labelling, an ROI (region of interest) line corresponding to the mucosa was drew; then the area of interest was identified setting up the threshold; lastly, the corresponding pixels^2^ were calculated using analyse toll; for c‐Kit‐ and Cx43‐labelled structures, the entire section was commuted to 8‐bit greyscale, then the quantification followed the steps described above. The mucosal integrity and inflammation degree were evaluated by two observers (CB, MGV) blind to each other, by a semi‐quantitative scoring system adapted from^15^ and using the following four criteria: loss of crypt architecture (graded 0–3, normal, mild, moderate, severe); extent of inflammatory cells infiltrate in lamina propria (graded 0–3, normal, mild, moderate, severe); goblet cell depletion (graded 0–3, normal, mild, moderate, severe), loss of muscularis mucosa continuity (graded 0–1, presence–absence). Thus, for each section, a numerical score of 0–10 was assigned. The count of labelled neurons was done by the two observers (CB, MGV) in the myenteric ganglia along the entire transverse section (2 sections/animal; 4 animals/group). The results were expressed as total number/section of NeuN or ChAT‐positive neurons and as percentage of ChAT labelled neurons per NeuN labelled neurons per section. Only neurons with a NeuN labelled nucleus surrounded by ChAT‐labelling were included in the count, as double labelled neurons. Amplitude of the spontaneous contractile activity was expressed as absolute values (grams) and measured when the maximal amplitude was reached. Contractions to methacholine were measured 30 s after a stable plateau phase was reached. All the results were reported as value ± SEM. Statistical analysis was performed by Student's *t*‐test or ANOVA as appropriate. When ANOVA indicated significant differences, we performed multiple comparisons between groups by Newman–Keuls post‐hoc test or Bonferroni's post‐hoc test. *p* < 0.05 was considered significant. The number of the specimens is designated by *n* in the results.

## 3 RESULTS

### 3.1 Body weight, food, water and kaolin intake

**FIGURE 1:**
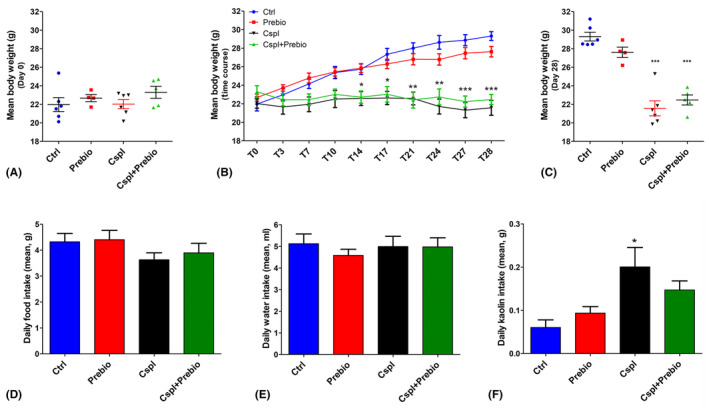
(A) The body weight was measured from day T‐4 to T0. The results were similar among all the groups. (B) Time‐course of the body weight gain during the 28 days of treatment. With time the Cspl‐ and Cspl+Prebio‐treated mice did not show any weight gain contrary to the Ctrl and Prebio mouse groups. (C) The body weight before the sacrifice was significantly lower than in Cspl and Cspl+Prebio mice. Daily food (D) and water (E) intake were similar in all groups during the treatment. (F) The kaolin intake during the treatment was significantly higher for the Cspl mice compared to all the other groups. Data are expressed as mean ± SEM. B, C = *p < 0.05, **p < 0.005, ***p < 0.001 vs. Ctrl and Prebio mice. F = *p < 0.05 vs. all the other groups (ANOVA, post hoc Newman–Keuls test).

The mouse body weight before the beginning of treatment (day 0) displayed no significant difference among the groups (Ctrl 21.97 ± 0.75, Prebio 22.67 ± 0.38, Cspl 22.02 ± 0.49, Cspl+Prebio 23.30 ± 0.65, Figure 1A). The time course (Figure 1B) showed a constant and continuous increase in Ctrl and Prebio groups; at the end of the treatment (day 28), the mean weight of these two groups was 29.31 ± 0.47 g and 27.62 ± 0.56 g, respectively, and these values were not significantly different (Figure 1B,C). Conversely, Cspl and Cspl+Prebio groups showed no significant weight gain during 4 weeks and, at the end of the treatment, their weight was 21.56 ± 0.80 g and 22.46 ± 0.54 g, respectively (Figure 1B,C). These values overlapped those recorded at the beginning of the treatment and were significantly lower with respect to Ctrl and Prebio groups (Figure 1A,C).

The mean quantities of the daily food intake and drinking water assumption during the treatment were not significantly different among the groups (Figure 1D,E). In contrast, the Cspl group consumed significantly more kaolin than the Ctrl and Prebio groups (Figure 1F) as an attempt to buffer drug induction of pica. Interestingly, the Cspl+Prebio group showed a value of kaolin intake intermediate between the Cspl and the other two groups. The statistical analysis, however, disclosed no significant differences with respect to the other groups (Figure 1F).

### 3.2 Stool production

**FIGURE 2:**
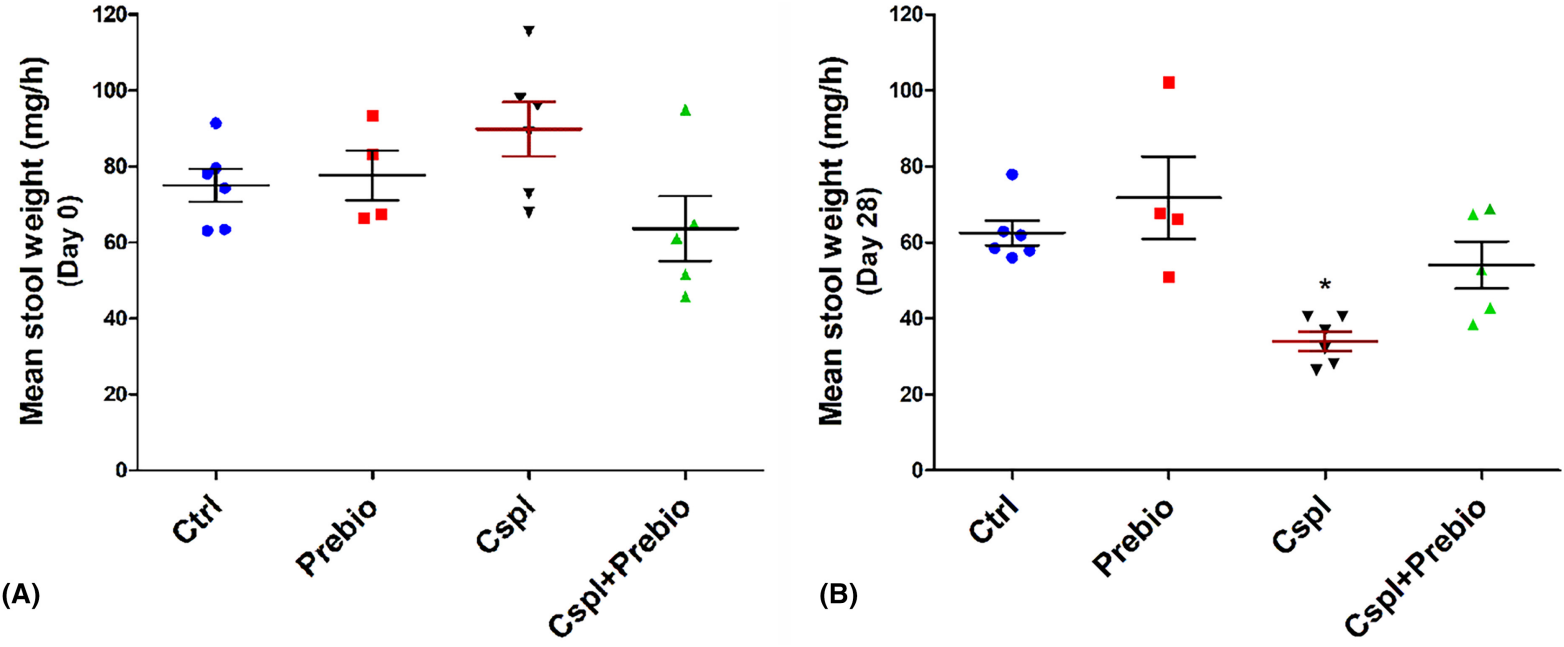
Stool production. (A) The faecal production the day before the beginning of the treatment was similar among the four groups of mice. (B) At the end of the treatment (28th day), the Cspl group showed a significant decrease in faecal production vs. all the other groups. Data are expressed as mean ± SEM. *p < 0.05 vs. all the other groups (ANOVA, post hoc Newman–Keuls test).

The quantity of stool production/hour was similar among the experimental groups before the beginning of the treatment (Figure 2A). At the end of the treatment, the stool production was significantly lower in the Cspl group compared with all the other three groups (Figure 2B).

### 3.3 Histology and histochemistry

#### 3.3.1 Haematoxylin and eosin and toluidine blue (TB) staining

**FIGURE 3:**
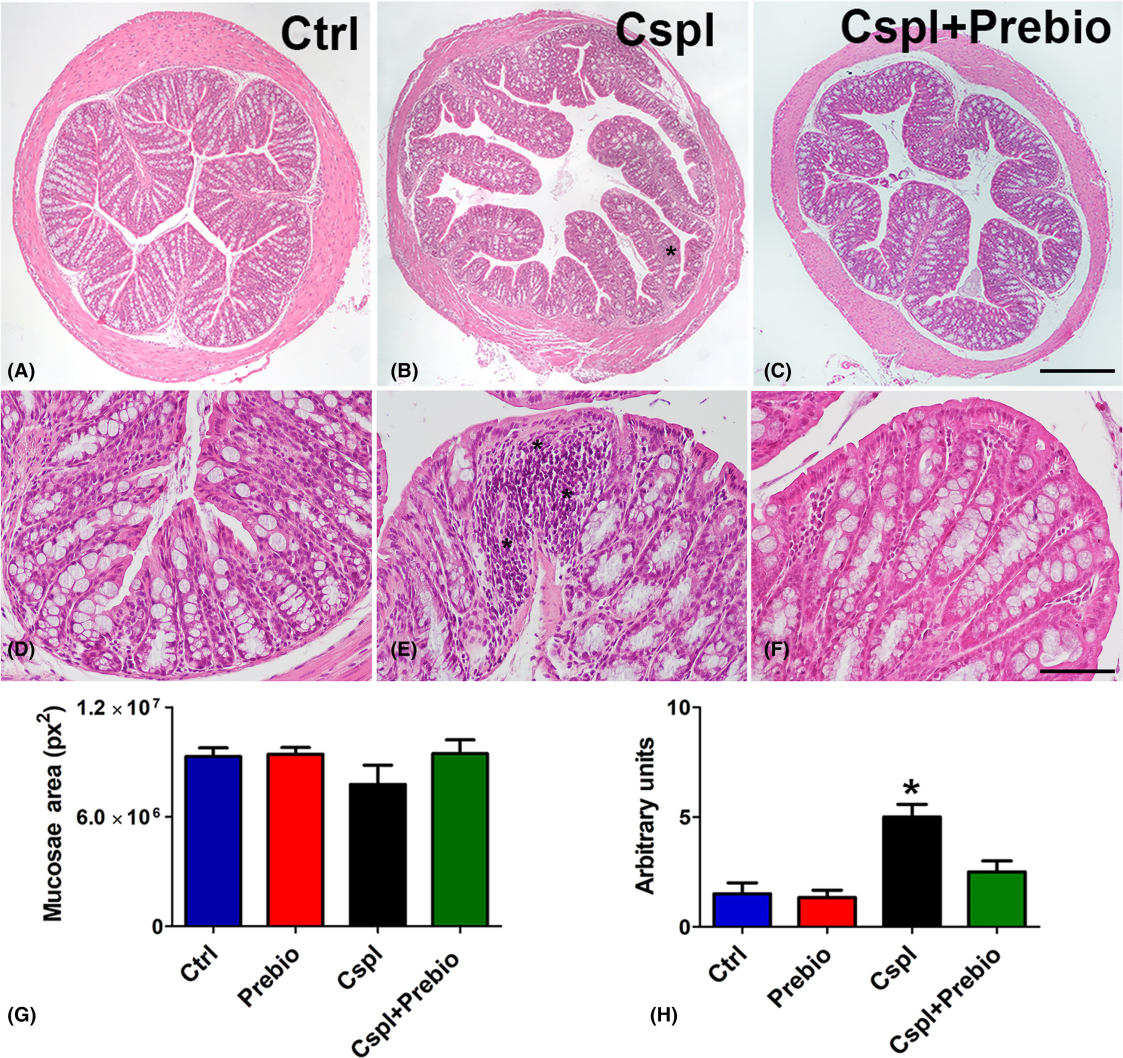
Haematoxylin and eosin staining. Ctrl (A–D), Cspl (B, E), Cspl+Prebio (C, F) mouse groups. Note the thinness of the villi and the dilatation of the crypts in Cspl‐treated mice compared with the other two groups of animals. Bar (A–C) = 200 μm; (D–F) = 50 μm. Asterisks indicate inflammatory cell infiltrates in the mucosa. Quantitation of the mucosae area (G) showed no difference among the groups of mice. Score analysis (H) was performed as described in Materials and Methods. Values are the median‐interquartile range. *p < 0.05 vs. all the other groups (ANOVA, Kruskal–Wallis, post hoc Newman–Keuls's test).

Haematoxylin and eosin staining (Figure 3A–F) of the transverse sections showed substantial integrity of the submucosa and muscle wall in all the groups of mice. In Cspl‐treated animals, the villi were often thinner, the crypts dilated (Figure 3B) and the lamina propria exhibited an increased inflammatory cell infiltrate, mostly made by lymphocytes (Figure 3B,E, asterisk) compared to Ctrl ones (Figure 3A,D). Quantitation of mucosal area showed a reduction in the Cspl group that did not reach the significance (Figure 3G). Quantification of mucosal damage using a semi‐quantitative composite score revealed that cisplatin‐induced a significant increase in the score compared to the Ctrl group (Figure 3H).

**FIGURE 4:**
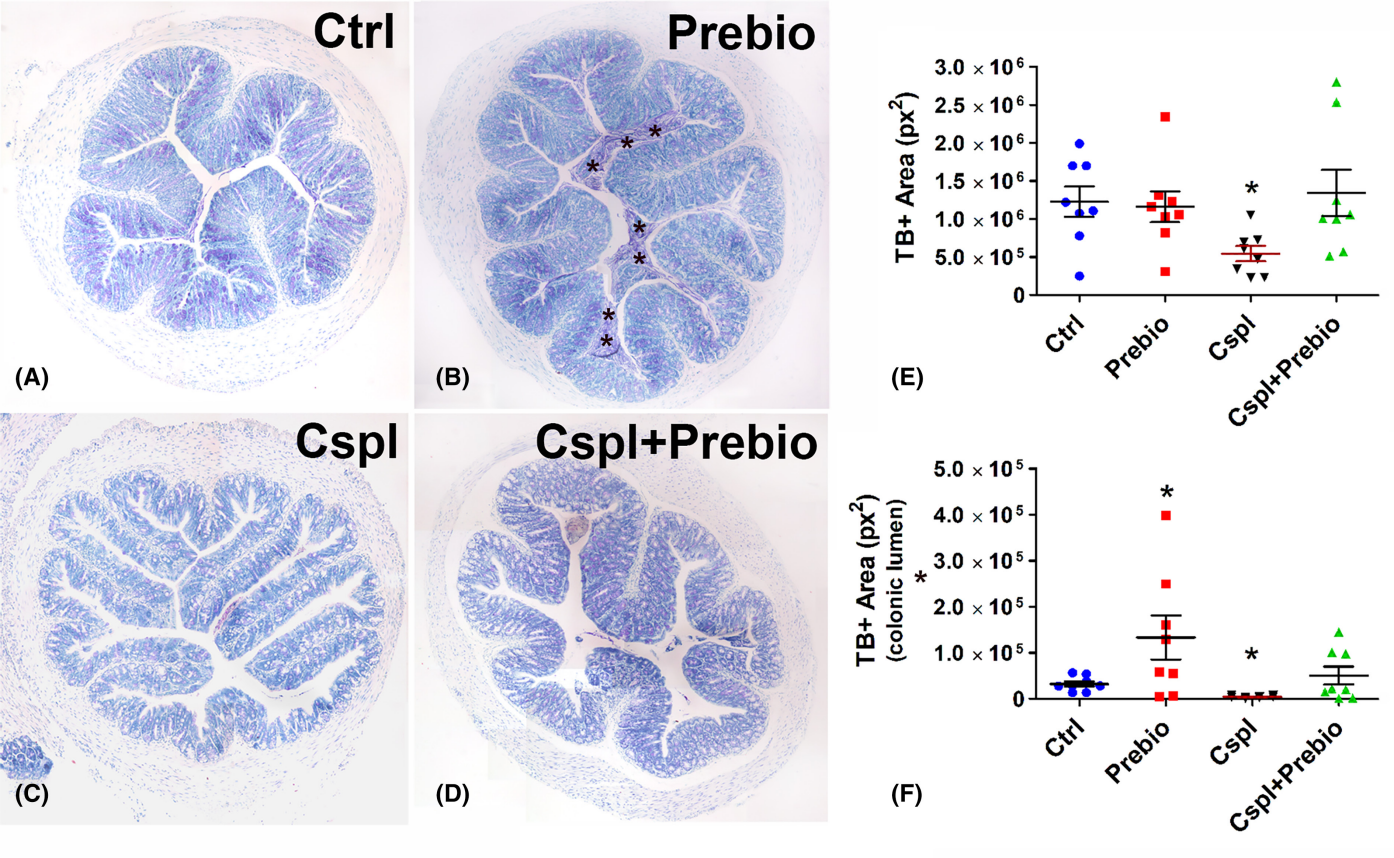
Toluidine blue staining. (A–D) The dye stained the goblet cells and the glandular cells in the crypts in all groups of mice. In Prebio group (B), the colonic lumen was filled with TB+ mucous secretion (asterisks). (E, F) Bar = 200 μm. Quantitation of TB+ material was significantly decreased in the epithelium (E) and in the lumen (F) of the Cspl group and significantly increased in the lumen of the Prebio group (F). *p < 0.05 vs. all the other groups (ANOVA, post hoc Newman–Keuls test).

TB staining was located in the goblet cells along the epithelium and in the glandular crypts (Figure 4A–D). In the Prebio group, a TB+ material was abundant in the colonic lumen (Figure 4B). Quantitation of the TB+ area in the epithelium and in the lumen showed a significant decrease in the Cspl group with respect to the other groups (Figure 4E) on the contrary, TB+ material in the colonic lumen was significantly increased in the Prebio group with respect to all the other groups (Figure 4F).

### 3.4 Immunohistochemistry

#### 3.4.1 c‐Kit‐ and Connexin (Cx)43‐immunoreactivity (IR)

**FIGURE 5:**
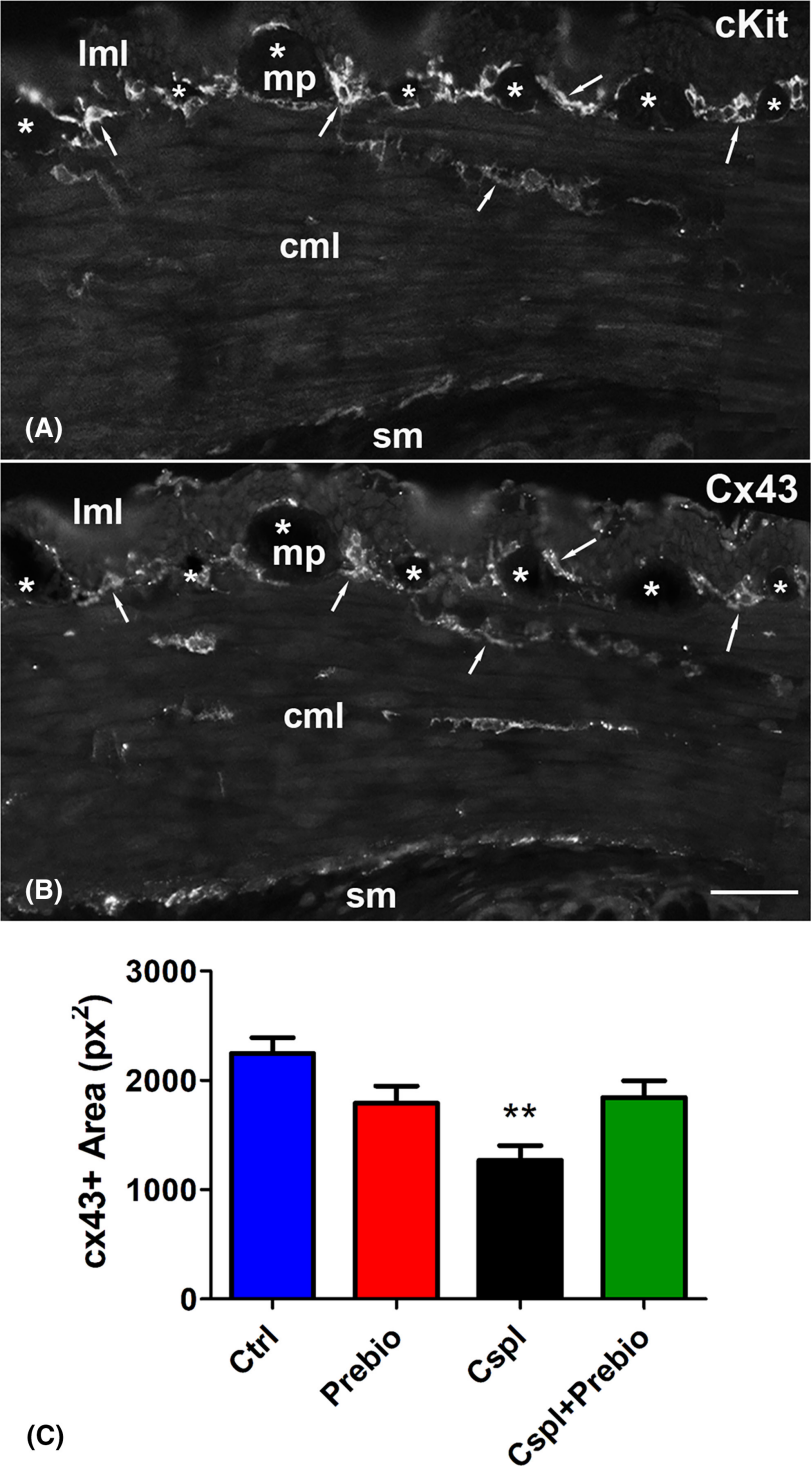
c‐Kit‐ and Cx43‐immunoreactivity (IR). (A) c‐Kit‐IR was detected in the ICC at the myenteric plexus (MY‐ICC), in muscle layers (IM‐ICC) and at the border with the submucosa (SM‐ICC). (B) Cx43‐IR overlapped that of c‐Kit. (A, B) The arrows highlight some of the several sites of overlapping between the two markers. The asterisks indicate the myenteric ganglia. Bar = 50 μm. (C) Quantitation of the Cx43 labelling showed a significant decrease in the Cspl‐treated group. cml, circular muscle layer; lml, longitudinal muscle layer; mp, myenteric plexus; sm, submucosa. **p < 0.005 vs. all the other groups (ANOVA, post hoc Newman–Keuls test).

c‐Kit‐IR was detected in myenteric plexus ICC (MY‐ICC), intramuscular ICC (IM‐ICC) and, at lower intensity, in the ICC located at the submucosal border of the circular muscle layer (SM‐ICC) (Figure 5A). Cx43‐IR was detected on the plasma membrane of elongated and branched cells corresponding to the ICC. All three types of ICC showed Cx43‐IR and the labelling intensity was comparable among them (Figure 5B). Quantitation of the Cx43‐IR showed a significant decrease in the Cspl group respect with all the other groups (Figure 5C). Quantitation of the c‐Kit‐IR gave similar results among the groups (data not shown).

#### 3.4.2 Choline acetyl‐transferase (ChAT)‐ and neuronal nuclei (NeuN)‐IR

**FIGURE 6:**
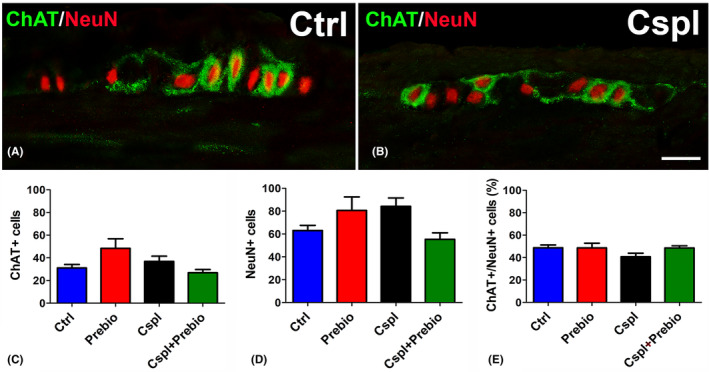
Choline Acetyl‐Transferasi (ChAT, green)‐ and Neuronal Nuclei (NeuN, red)‐IR. (A, B) NeuN‐IR was detected in the nuclei of several myenteric neurons. Some of them showed the ChAT labelling in the cytoplasm and processes. Bar = 20 μm. Quantitation of the total number of NeuN‐ and ChAT‐IR neurons (C, D) and of the percentage of the cholinergic ones with respect to the total myenteric neurons (E) showed no differences among the four groups.

The pan‐neuronal marker NeuN‐IR was exclusively located in the nuclei of the myenteric neurons, whereas the ChAT‐IR was observed in the cytoplasm of several myenteric neurons and in a few intramuscular nerve fibres (Figure 6A,B). The quantitation of NeuN‐ and ChAT‐IR neurons displayed no significant differences among the groups (Figure 6C–E).

### 3.5 Functional studies

#### 3.5.1 Spontaneous mechanical activity

**FIGURE 7:**
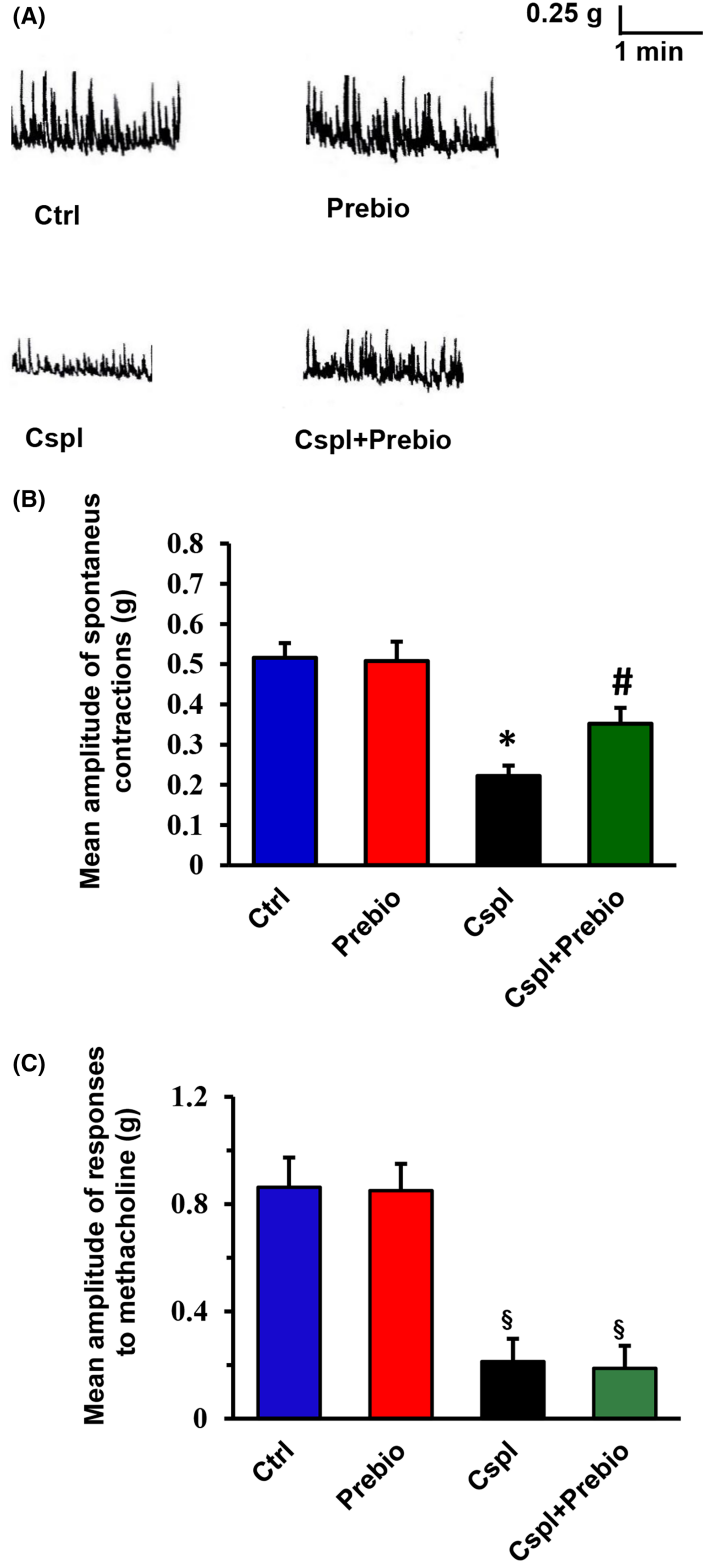
(A) Typical tracings showing the spontaneous mechanical activity recorded in preparations from the different animal groups. In strips from Prebio‐treated animals (right‐hand upper trace), the amplitude of the spontaneous contractions was not different with respect to that obtained in the Ctrl mice (left‐hand upper trace). The amplitude of the spontaneous contractions was significantly reduced in preparations from Cspl‐treated animals (left‐hand lower trace). In strips from Cspl+Prebio‐treated mice (right‐hand lower trace), the amplitude of the spontaneous contractions was significantly increased with respect to Cspl mice. The motility pattern showed no differences among preparations from the four animal groups. (B) Bar chart showing the mean amplitude of the spontaneous contractions in preparations from the different animal groups. No statistical differences in the amplitude of the spontaneous contractions were present between preparations from Ctrl and Prebio mice. Note the great reduction in amplitude of the spontaneous contractions in strips from Cspl mice and its partial recovery in Cspl+Prebio‐treated mice. (C) Bar chart showing the mean amplitude of the direct muscle contractions elicited by methacholine in preparations from the different animal groups. No statistical differences were revealed between preparations from Ctrl‐ and Prebio‐treated mice. Note the great reduction in amplitude of the response to methacholine in strips from Cspl‐ and Cspl+Prebio‐treated mice. All values are means ± SEM of 5–8 preparations. *p < 0.05 vs. all the other groups; #*p* < 0.05 vs. Cspl mice ^§^ *p* < 0.05 vs. Ctrl and Prebio‐treated mice (ANOVA, post hoc Bonferroni's test).

Colonic preparations from Ctrl (*n* = 8) mice exhibited spontaneous contractile activity, consisting of rhythmic changes in isometric tension (mean amplitude, 0.51 ± 0.03 g) (Figure 7A,B). Addition of TTX (1 × 10^−6^ M) to the bath medium (*n* = 3) did not affect the motility pattern and the amplitude of the spontaneous contractions (mean amplitude, 0.54 ± 0.05 g), indicating their muscular nature.

Strips from Prebio mice (*n* = 6) showed the same motility pattern as Ctrl animals (Figure 7A,B) (mean amplitude, 0.50 ± 0.04 g). In preparations from Cspl mice (*n* = 8), the motility pattern did not differ from that observed in strips from the other groups (Figure 7A,B), whereas the amplitude of the spontaneous contractions was significantly reduced (mean amplitude, 0.22 ± 0.2 g) (Figure 7A,B).

In strips from Cspl+Prebio mice (*n* = 6), the motility pattern showed no differences with respect to that observed in preparations from all the other animal groups (Figure 7A,B). However, the amplitude of the spontaneous contractions was statistically greater (mean amplitude, 0.35 ± 0.03 g) with respect to that observed in strips from Cspl mice (Figure 7A,B) but still statistically reduced compared to that of Ctrl and Prebio mice (Figure 7A,B). In order to prove that all the above‐reported effects were muscular in origin, the direct smooth muscle responses to methacholine were evaluated. Addition of the muscarinic agonist methacholine to the bath medium caused a sustained contracture whose amplitude was greatly reduced in strips from Cspl (*n* = 5) and Prebio+Cspl (*n* = 5) in respect to Ctrl (*n* = 5) and Prebio (*n* = 5) animal groups (Figure 7C).

## 4 DISCUSSION

The present findings show that cisplatin, administered in mice at a dosage comparable to that used in humans,^15^ induced several behavioural, morphological and functional gastrointestinal changes. The daily control of the physiological parameters indicated a progressive deterioration of the general conditions consisting in the lack of weight gain and in a eating behaviour characterized by a greater intake of kaolin, both signs of pica. At the end of the treatment, the mice showed a significant decrease in stool production, a decreased mucus secretion and signs of mucosal inflammation in the proximal colon, that is, the colonic region we examined. Daily administration of prebiotics prevented alterations in stool production, mucus secretion and inflammation and significantly reduced kaolin intake while having no effect on the lack of weight gain. Functional studies showed a significant depression in amplitude of both spontaneous and methacholine‐induced muscular contractions in colonic strips of Cspl treated mice. Immunohistochemistry evidenced no neuronal loss but a reduced Cx43 expression in the ICC. Prebiotics treatment partially prevented the alterations of spontaneous contractions and of Cx43 expression while had no effect on methacholine responses.

In accordance with previous investigations, chronic cisplatin impaired the animal's growth.^15^, ^16^ Starting from the first week of treatment, regardless of the presence of prebiotics in the diet, the weight gain showed a halt which lasted until the end of the fourth week. Failure to gain weight is probably attributable to mucositis. This condition is the most common finding following cisplatin treatment, and the upper GI regions (stomach, ileum) are particularly susceptible to the drug toxicity.^3^, ^14^, ^15^, ^16^ Stomach injury, in particular, would play a major role. Using a dose lower than the current one, we found a remarkable fundus distention, mucosal damage and neuropathy.^15^ Functional studies showed a significant delay of gastric emptying responsible for nausea and pica.^13^, ^14^ Likewise, comparable or lower doses of cisplatin caused ileum injury that compromises the food absorption.^3^, ^13^, ^14^, ^19^ Thus, because of the gastric and ileal damages, our animals did not growth although they assumed as much food as controls.

The reduction in mucin production in the proximal colon of Cspl‐treated mice might be due to the drug antimitotic activity with reduced cell renewal or/and impairment of mucin synthesis by surviving glandular cells.^33^ Our finding probably depends on both conditions as the decrease in TB+ area was accompanied by a decrease in PAS+ cells (personal observation). In the colon, unlike the ileum, the mucus forms a continuous and thick stratum upon the epithelium and has two important functions: it protects the epithelium reinforcing the intestinal barrier and nourishes the microbiota. Thus, its reduction favours the mucosal damage, the dysbiosis and the appearance of mucositis.^10^, ^12^, ^16^, ^34^ The efficacy of prebiotics to prevent mucus secretion decrease might be due either to their ability to interact with soluble molecules present in the mucus avoiding their degradation and loss and/or to select bacterial strains productors of molecules able to recruit myeloid cells which, in turn, stimulate mucin synthesis.^10^, ^12^, ^35^

The significant reduction of stool production found in the Cspl group at the end of the treatment agrees with the finding recorded in the functional study. The motility pattern of the colon exhibits very complex features and a variety of patterns have been described differing in relation to animal species^36^, ^37^ and colonic segments.^38^, ^39^ Presently, the strips of proximal colon exhibit a spontaneous contractile activity consisting of rhythmic changes in isometric tension. While no differences in motility patterns were observed among all the mice groups, a great reduction in the amplitude of the spontaneous contractile activity was observed in strips from the Cspl‐treated mice. Co‐treatment with prebiotics partially counteracted this reduction and prevented the stool production decrease. The amplitude of the muscular spontaneous contractions did not differ between strips from Ctrl and Ctrl+Prebio mice indicating that prebiotics pretreatment ‘per se’ had no effects on the spontaneous contractile activity. Impaired intestinal motility had already been reported, either in rats or in mice, following chronic treatment with cisplatin and attributed to a neuropathy consisting of a reduction in the number of myenteric neurons.^14^, ^15^, ^16^, ^19^ In our specimens, however, no change in the total number of myenteric neurons was detected. Therefore, we turned our attention to other cell types, such as the ICC, as possible targets of Cspl damage. In the colon, these cells generate spontaneous contractions and mediate a complex synchronization of excitation and inhibition of the smooth muscle syncytium.^40^, ^41^, ^42^ In the colon the three ICC subpopulations, all c‐Kit positive, through gap junctions form a 3D network along the entire muscle wall and contact smooth muscle cells controlling their activity; finally, ICCs receive innervation through synapse‐like contacts.^21^ A significant decrease of c‐Kit expression in the ICC of the gastric antrum of Suncus Murinus was reported using a sublethal dose of cisplatin in a single injection,^26^ while a c‐Kit mRNA increase was found in the ileum of mouse receiving chronic cisplatin.^19^ To date, ICC has never been studied in the colon of rodents treated with cisplatin. Therefore, by using immunohistochemistry, we looked for potential ICC changes related to the altered amplitude of the motor pattern observed in Cspl mice. The results showed no change in c‐Kit labelling among the groups while a significant reduction of the gap‐junction protein Cx43, one of the main proteins forming connexons, was detected. To note that whereas some papers describe Cx43 as the common connexin of both, smooth muscle cells and ICC gap junctions,^43^, ^44^, ^45^ others reported a selective Cx43 labelling of the ICC in human bowel.^46^, ^47^, ^48^ In our samples of mouse proximal colon, Cx43 labelling was present at the level of the MP and, to a lesser extent, at the border of the SM and in the thickness of the circular muscle layer; the labelling distribution markedly overlapped with that of the three ICC subtypes, as confirmed by the c‐Kit antibody labelled twin slices.

The reduction in the Cx43 might be due to a reduced number of connexons and/or to altered protein composition of these channels. In both cases, the consequence is an impairment in the electrical transmission to the smooth muscle. The ability of prebiotics to prevent the Cx43 loss suggests the involvement of the microbiota. In regard, it was reported, in humans and mice, that gut inflammatory processes of different genesis trigger a molecular cascade able to affect the ICC, causing ICC‐pathy^49^, ^50^ and that fructans prebiotic such as inulin, that represents the major component of our prebiotic mixture, has anti‐inflammatory and antioxidant properties besides promoting Bifidobacterium increase.^35^, ^51^

Functional results in Cspl‐treated mice showed decreased amplitude of both TTX‐insensitive spontaneous contractile activity and direct muscle contractile response to methacholine suggesting an impairment of the smooth muscle. The ability of Cspl to influence the response to methacholine does not ‘per se’ exclude an involvement of the cholinergic pathways. However, since the number of ChAT‐IR myenteric neurons was found to be not modified in Cspl‐treated animals, we hypothesize that the drug, besides indirectly affecting the contractility of smooth muscle cells (due to the ICC‐pathy), also causes direct damage to muscarinic receptors. The prebiotics were ineffective in preventing the reduction of the response to methacholine.

In conclusion, the present study on mice shows that Cspl injured proximal colon. Beyond the lack of weight gain, due to systemic GI damage, in the proximal colon, the drug impairs mucin production and mucosa integrity causing mucositis and determines a consistent reduction in amplitude of spontaneous contractions associated with constipation. These latter manifestations are attributable to an ICC‐pathy characterized by a significant decrease of Cx43 expression and, perhaps, to postsynaptic muscarinic damage as shown by the deficit of the contractile response to the cholinergic agonist methacholine. Prebiotic mixture administered in chronic during chemotherapy, prevented the mucositis and the loss of mucins, counteracted the loss of Cx43 and the spontaneous motility impairment, but had no effect on the postsynaptic muscarinic receptor alteration. Based on our results, we can deduce that the beneficial effects of prebiotics on the intestinal wall are the result of their protective action on the microbiota, mostly due to the mucous layer preservation.

Therefore, the proposed treatment, which is easy to take and free of side effects, could guarantee the maintenance of the effective dose of chemotherapy by preventing and/or reducing its toxicity and even improving its effectiveness.

## AUTHOR CONTRIBUTIONS

**Cristina Biagioni:** Data curation (equal); formal analysis (equal); investigation (equal); methodology (equal); software (equal); visualization (equal). **Chiara Traini:** Conceptualization (supporting); data curation (equal); formal analysis (equal); investigation (equal); methodology (equal); software (equal); supervision (equal); validation (equal); visualization (equal); writing – review and editing (equal). **Maria Simonetta Faussone‐Pellegrini:** Visualization (equal); writing – review and editing (equal). **Eglantina Idrizaj:** Data curation (equal); formal analysis (equal); investigation (equal); methodology (equal); software (equal); visualization (equal); writing – review and editing (equal). **Maria Caterina Baccari:** Data curation (equal); methodology (equal); supervision (equal); validation (equal); visualization (equal); writing – review and editing (equal). **Maria Giuliana Vannucchi:** Conceptualization (lead); data curation (equal); formal analysis (equal); funding acquisition (lead); methodology (equal); project administration (lead); supervision (equal); validation (equal); visualization (equal); writing – original draft (lead); writing – review and editing (equal).

## FUNDING INFORMATION

This research was supported by funds from the University of Florence ex‐60% (RICATEN22) to MGV.

## CONFLICT OF INTEREST STATEMENT

The authors declare no conflict of interest.

## Supporting information


Table S1

## Data Availability

Data available on request from the authors.
