# Supplementary material for: Prebiotics counteract the morphological and functional changes secondary to chronic cisplatin exposition in the proximal colon of mice
Source: J Cell Mol Med. 2024 Mar 6;28(6):e18161. doi: 10.1111/jcmm.18161 (PMC10915824; doi:10.1111/jcmm.18161)
Supplement: Supplementary file 1 — Table S1 [file JCMM-28-e18161-s001.docx]

Table S1. Primary and secondary antibodies

| **Primary antibody** | **Host** | **IHC** | **Conjugated** | **Producer** |
| --- | --- | --- | --- | --- |
| Anti-NeuN | Mouse | 1:100 | No | MAB377, Millipore Corporation, CA, USA |
| anti-ChAT | Goat | 1: 200 | No | AB144P; EMD Millipore Corporation, CA, USA |
| Anti-CD117, c-Kit | Rabbit | 1: 200 | No | A4502; Dako Agilent, CA, USA |
| Anti Cx43 | Rabbit | 1:100 | No | 3512; Cell Signaling Technologies, MA, USA |
| **Secondary antibody** | **Host** | **IHC** |  | **Producer** |
| Anti-Goat | Donkey | 1:333 | AlexaFluor ® 488 | A11055; RRID AB_2534102, Thermo Fisher Scientific. |
| Anti-mouse | Donkey | 1:333 | Alexa Fluor® 594 | 715-585-151; Jackson Laboratories, West Grove, PA, USA |
| Anti-rabbit | Goat | 1:333 | AlexaFluor ® 488 | 111-547-003; Jackson Laboratories, West Grove, PA, USA |
